# Development of a fast and low-cost qPCR assay for diagnosis of acute gas pharyngitis

**DOI:** 10.1186/s12941-016-0162-0

**Published:** 2016-08-08

**Authors:** Mustafa Kolukirik, Mesut Yılmaz, Orhan Ince, Canan Ketre, Ayşe Istanbullu Tosun, Bahar K. Ince

**Affiliations:** 1 ENGY Environmental and Energy Technologies Biotechnology Research and Development Limited Company, Istanbul, Turkey; 2 Infectious Diseases and Clinical Microbiology, Istanbul Medipol University, Istanbul, Turkey; 3 Istanbul Technical University, Istanbul, Turkey; 4 Microbiology, Istanbul Medipol University, Istanbul, Turkey; 5 Bogazici University, Istanbul, Turkey

**Keywords:** Group A streptococci, Acute pharyngitis, qPCR, Rapid antigen detection test

## Abstract

**Background:**

Group A streptococci (GAS) are the most common bacterial cause of acute pharyngitis and account for 15–30 % of cases of acute pharyngitis in children and 5–10 % of cases in adults. In this study, a real-time quantitative PCR (qPCR) based GAS detection assay in pharyngeal swab specimens was developed.

**Methods:**

The qPCR assay was compared with the gold standard bacterial culture and a rapid antigen detection test (RADT) to evaluate its clinical performance in 687 patients. The analytical sensitivity of the assay was 240 cfu/swab. Forty-five different potential cross-reacting organisms did not react with the test. Four different laboratories for the reproducibility studies were in 100 % (60/60) agreement for the contrived GAS positive and negative swab samples.

**Results:**

The relative sensitivities of the RADT and the qPCR test were 55.9 and 100 %; and the relative specificities were 100 and 96.3 %, respectively. Duration of the total assay for 24 samples including pre-analytical processing and analysis changed between 42 and 55 min depending on the type of qPCR instrument used. A simple DNA extraction method and a low qPCR volume made the developed assay an economical alternative for the GAS detection.

**Conclusion:**

We showed that the developed qPCR test is rapid, cheap, sensitive and specific and therefore can be used to replace both antigen detection and culture for diagnosis of acute GAS pharyngitis.

## Background

Acute pharyngitis is a nonspecific symptom that can result from a number of viral or bacterial infections. Group A streptococci (GAS) are the most common bacterial cause of acute pharyngitis and account for 15–30 % of cases of acute pharyngitis in children and 5–10 % of cases in adults [1]. The cost per case of GAS pharyngitis was estimated to be approximately $205, with about half of the costs attributed to nonmedical costs such as missed days of work by parents for child care [2].

GAS pharyngitis is usually self-limited and resolves without the need for antibiotic treatment [3]; however, a minority of patients develop severe complications such as scarlet fever and peritonsillar cellulitis, as well as immune-mediated complications, including poststreptococcal glomerulonephritis and acute rheumatic fever. Early treatment of GAS pharyngitis with appropriate antibiotics is known to reduce symptom severity and duration, decrease transmission of the organism, and reduce the risk of acute rheumatic fever [4]. Incidence of the rheumatic heart disease changes between 0.03 and 2.1 % based on the development status of the countries [5].

As most pharyngitis is viral in origin, accurate diagnosis can reduce the unnecessary use of antibiotics and potential development of antibiotic resistance [6, 7]. However, accurate diagnosis of GAS pharyngitis is difficult for a number of reasons. First, diagnosis of GAS pharyngitis using clinical signs alone is unreliable due to the broad overlap in symptoms between the viral and bacterial etiologies [4]; physicians miss up to 50 % of GAS pharyngitis cases and identify 20–40 % of non-GAS sore throat cases as requiring antibiotics [8]. Second, many children are asymptomatic carriers of GAS, with the prevalence of GAS throat carriage estimated at 12 % [9]. Third and the most important, the standard procedure for laboratory detection of GAS, culture on blood agar, typically requires 24–48 h [10], which is problematic for physicians to provide the diagnosis on the same day of patients office visit.

Value of rapid testing for the GAS pharyngitis diagnosis in directing clinical management and reducing unnecessary antibiotic prescription has been documented [11, 12]. The fast diagnosis methods include rapid antigen detection tests (RADTs) and nucleic acid-based methodologies. RADTs provide results within minutes, but exhibit only 72–90 % sensitivity compared to that of a culture [13]. Polymerase chain reaction (PCR) [14, 15], real-time quantitative PCR (qPCR) [16, 17] and loop-mediated isothermal amplification (LAMP) [18, 19] based tests for the detection of GAS have been described. These tests have high sensitivity (>93 %) and good specificity (>95 %). The PCR and qPCR based tests have not been found wide application due to insufficient clinical evaluation [14, 16], high cost [17] and labor intensive laboratory work [15]. A LAMP based method, the Illumi-gene group A Streptococcus assay, has been developed by Meridian Bioscience and cleared by the FDA. Henson et al. [19] and Anderson et al. [18] evaluated its clinical performance and reported that the assay is rapid (<1 h), easy to perform and highly sensitive and specific.

Current Infectious Diseases Society of America (IDSA) guidelines state that a clinical diagnosis of GAS pharyngitis must be confirmed [4]. Reimbursement for the culture and the molecular based analyses are 9.12$ and 48.24$ respectively in USA, which makes the Illumi-gene group A Streptococcus assay (28$/test), an economically feasible tool for diagnosis of GAS pharyngitis (Sales Sheet: Illumigene Group A Streptococcus). On the other hand, there is no reimbursement for the molecular detection of GAS pharyngitis in most of the developing countries due to the very high costs of the tests. Furthermore, the clinical signs have been the major diagnostic tools in developing countries like Turkey where the cost of antibiotic treatment is very low (3–10$). This has resulted in considerable social burden [20] and unnecessary antibiotic prescriptions in up to 75 % of patients with acute tonsillopharyngitis [21] and a total (outpatients and hospital care) antibiotic use of 42.3 defined daily doses/1000 inhabitants per day (DID) for Turkey which has made Turkey the first among 40 countries in Europe in the calculation of unit ranks for antibiotic usage [22].

In this study, we aimed to develop a qPCR based GAS detection assay in pharyngeal swab specimens that is as rapid as the previous molecular tests, as sensitive and specific as the culture test and as economical as the low-cost antibiotic treatments.

## Methods

### Study participants and collection of specimens

The study was conducted in accordance with the Declaration of Helsinki and approved by Istanbul Medipol University (IMU) Research Ethics Committee. The throat swab specimens were collected at IMU Hospital from 687 patients aged between 5 and 12 years presenting acute sore throat over the winter/spring of 2012 and 2013. The study population included 356 (51.8 %) female and 331 (48.2 %) male patients. No restrictions were placed on gender, medications or known pharmaceutical therapies.

Two blind samples were collected using the BBL CultureSwab EZ II—Double Swab (Becton–Dickinson, USA) according to the standard methods [4]. One swab was used for the RADT and culture and the other was used for the qPCR assay. The samples for the culture and antigen tests were transported to Istanbul Medipol University Laboratory at ambient temperature and processed on the day of sample collection. The samples for the molecular analysis were transported to Istanbul Technical University Molecular Biology, Genetics and Biotechnology Research Center (ITU MOBGAM) at +2 to 8 °C and analyzed on the day of sample collection.

### Culture and antigen test

The swabs were inoculated on BBL strep selective agar (Becton–Dickinson, USA) plates and incubated at 35 °C for 16–18 h. Catalase negative colonies with a morphology of β-hemolytic streptococcus were transferred to 5 % sheep blood agar and subjected to bacitracin, sulfamethoxazole–trimethoprim (SXT) susceptibility test, BBL DrySlide PYR and BBL Streptocard Enzyme Latex Test Kit (Becton–Dickinson, USA) for GAS confirmation.

After inoculation of the SSA plate, the same swab was used for evaluation with the Clearview^®^ Strep A Exact II Cassette test (Inverness Medical Professional Diagnostics, USA). The procedure was performed according to the instructions provided by the manufacturer.

### Molecular analyses

The swabs were cut and placed into 1.5 ml microcentrifuge tubes containing 400 μl of 0.1 M Tris–HCl pH 8.0. The tubes were placed in TSS-2000 turbo thermal shaker & heater (Inovia Technology, Turkey), shaken for 20 s at 3000 rpm, incubated at +95 °C for 10 min and shaken for 20 s at 3000 rpm. The swabs were removed from the sample tubes and discarded. The samples were immediately used in qPCRs and stored at −20 °C after the qPCR set up.

Two different qPCRs (qPCR-GAS and qPCR-PC) were set up by combining 5 μl of the supernatant with 5 μl of two different 2x qPCR master mix in 0.1 ml reaction tubes. The qPCR-GAS and qPCR-PC contained primers targeting *Streptococcus pyogenes* pyrogenic exotoxin B (speB) and *Bacillus thermocatenulatus* triacylglycerol lipase (BTL2) genes respectively. The qPCR-PC also contained BTL2 gene. The qPCR-PC was set up as a positive control reaction to evaluate inhibitory effect of the sample on the DNA amplification. Five microlitre DNase free molecular grade water was combined with 5 μl of 2x qPCR-GAS master mix as a negative control (qPCR-NC).

Biospeedy EvaGreen Real-Time PCR 2x premix (Bioeksen R&D Technologies, Turkey) was used for qPCR. The premix contains 12 mg/ml bovine serum albumin (BSA), 40 mg/ml PEG 400, %0.5 Tween 20, 40 mM Tris–HCl pH 8.0, 100 mM KCl, 3 mM MgCl_2_, 0.4 mM dNTP mix, 0.2U Hot-Start Taq DNA Polymerase and 0.2x EvaGreen Dye. The 2x qPCR-GAS master mix was prepared by adding 200 nM of the each speB1166-F (5′-AAAGTAGGCGGACATGCCTTTG-3′) and speB1268-R (5′-CAAGACGGAAGAAGCCGTCAG-3′) primers to the premix. The 2x qPCR-PC master mix was prepared by adding 200 nM of the each BTL908-F (5′-CGACGGATACTGCCCGCTAC-3′) and BTL1014-R (5′-CCGTTCGGTGGAAAAGCTCA-3′) primers and 5 ng of their target BTL2 gene to the premix.

QPCR cycling conditions were an initial 3 min at 95 °C step, followed by 35 amplification cycles of 95 °C for 5 s and 60 °C for 25 s. A melt-curve analysis with a temperature transition rate of 0.5 °C/s was performed from 60 to 90 °C to determine if only one amplified product was generated during qPCR. The qPCR was carried out using four different Real-Time PCR system: Biorad CFX Connect (Bio-Rad Laboratories, USA), LightCycler 480 (Roche Applied Science, USA), StepOne Plus (Life Technologies, USA) and Xxpress (BJS Biotechnologies, UK). The samples which had a melting temperature (Tm) of qPCR-GAS between 80 and 81 °C and no specific amplification in qPCR-NC were considered positive. The samples that had no specific amplification in the qPCR-GAS and qPCR-NC were considered negative for GAS. Threshold cycle (Ct) of the qPCR-PC was lower than 25 with a Tm between 80 and 81 °C. The qPCR-PC Ct values higher than 25 were considered an indicator of PCR inhibitor interference.

The amplified DNA fragments in positive qPCR-GAS reactions from the tested 687 patients and the contrived positive (*S. pyogenes* ATCC 19615) swab samples were purified using High Pure PCR Product Purification Kit (Roche Applied Science, USA) and sequenced using the ABI prism Big Dye Terminator Cycle Sequencing Ready Reaction Kit on an ABI Prism 377 DNA sequencer (Life Technologies, USA). The sequences were analyzed in Chromas software package version 2.0 (Technelysium, Australia) and manually checked for the reading errors. The sequences were aligned with *S. pyogenes* strain ATCC 19615 whole genome region from 849512 to 849614 (Access# CP008926.1) coding speB gene using Clustal Omega (http://www.ebi.ac.uk/Tools/msa/clustalo/).

### Analytical sensitivity

Twenty replicates of the BBL CultureSwab EZ—Single Swab (Becton–Dickinson, USA) were spiked with 10^1^–10^4^ CFU *S. pyogenes* ATCC 19615 in 100 μl Buffer1 (0.1 M Tris–HCl pH 8.0). Following determination of a detection limit in a wide range, e.g. between 2 × 10^2^ and 3 × 10^2^ CFU, five more dilutions were prepared within this range. Limit of detection (LOD) stated a 95 % (19/20) probability of obtaining the reference samples positive for GAS. *S. pyogenes* ATCC 49399 and 12344 strains were also tested at the determined LOD. Interferences of 5 mg/mL mucus and 10 % v/v human saliva in Buffer1 were tested with contrived positive (ATCC 19615) samples prepared at the determined LOD. Analytical sensitivity studies were carried out using Biorad CFX Connect qPCR instrument.

### Analytical specificity

In-Silico PCR with speB1166-F and speB1268-R primers was carried out using the Primer Blast tool (http://www.ncbi.nlm.nih.gov/tools/primer-blast/) to test the cross reactivity among all available sequences in DNA databases. 55 bp fragment of *Bacillus* sp. GL1 unsaturated glucuronyl hydrolase gene (AB019619) and 1217 bp fragment of *Myxococcus stipitatus* DSM 14675 long-chain-fatty-acid-CoA ligase gene (CP004025) were amplified with total mismatches of 7 and 9 bases respectively. *Myxococcus stipitatus* DSM 14675 and chemically synthesized 55 base fragment of the *Bacillus* sp. GL1 DNA (Macrogen Inc., Europe) were included in the analytic specifity tests.

The BBL CultureSwab EZ—Single Swab (Becton–Dickinson, USA) were spiked with 10^6^ CFU bacterial or fungal organisms in 100 μl Buffer1. 20 ng/μl human DNA, 20 ng/μl *Bacillus* sp. GL1 DNA and the following organisms were tested for the cross reactivity:

*Acinetobacter baumannii, Aeromonas hydrophila, Arcanobacterium haemolyticum, Bordetella bronchiseptica, Burkholderia cepacia, Campylobacter jejuni, Candida albicans, Citrobacter freundii, Corynebacterium diphtheria, Corynebacterium pseudodiphtheriticum, Enterococcus faecalis, Enterococcus faecium, Escherichia coli, Haemophilus influenzae, Klebsiella oxytoca, Klebsiella pneumoniae, Lactococcus lactis, Legionella pneumophila, Listeria monocytogenes, Moraxella catarrhalis, Morganella morganii, Myxococcus stipitatus,Neisseria pharyngis, Neisseria meningitidis, Proteus mirabilis, Pseudomonas aeruginosa, Staphylococcus aureus, Staphylococcus epidermidis, Stenotrophomonas maltophilia, Streptococcus agalactiae, Streptococcus anginosus, Streptococcus bovis, Streptococcus canis, Streptococcus dysgalactiae (subspecies equisimilis), Streptococcus equinus, Streptococcus intermedius, Streptococcus mitis, Streptococcus mutans, Streptococcus pneumoniae, Streptococcus salivarius, Streptococcus suis, Streptococcus uberis, Streptococcus* sp. *viridans type.*

### Reproducibility

Contrived high positive (10^5^ CFU ATCC 19615), positive (240 CFU ATCC 19615), low negative (10^5^ CFU non-target organism mix) and negative (without any organism) swab samples were prepared using Buffer1. Five sets of blind-coded panels of 12 samples (three replicates of the four swab types) were supplied to four independent laboratories each having a different qPCR instrument (Biorad CFX Connect, LightCycler 480, StepOne Plus and Xxpress). Samples were randomly sorted within each panel to mask sample identities. Different operators at each site performed testing on the same day (intra-assay variability) for 5 days (inter-assay variability). Duration of the total assay for 12 samples was also measured each day.

### Statistical analysis

After assessment of normality of data, differences between the replicate samples in terms of Ct and Tm values were evaluated using the Student’s t test. Analyses were performed using the software MINITAB 17 (Minitab Ltd., England).

The sample size to show that the developed assay is not worse than the culture was calculated based on 5 % non-inferiority limit as described by Blackwelder [23]. It was estimated that “If there is a true 5 % difference between the developed assay and the culture, then 375 patients are required to be 90 % sure that the upper limit of a one-sided 95 % confidence interval (or equivalently a 90 % two-sided confidence interval) will exclude a difference in favour of the standard group of more than 5 %”.

## Results

LOD of the qPCR test was 240 CFU/Test for *S. pyogenes* ATCC 19615. The swabs containing 240 CFU *S. pyogenes* ATCC 49399 or 12344 strains also produced positive reactions. The qPCRs at the stated LOD were positive for all of the replicates with Ct value of 25.9 ± 1.2 and a single Tm peak at 80.3 ± 0.2 °C. The qPCR product sequences matched 100 % with *S. pyogenes* strain ATCC 19615 speB gene.

Human DNA, *Bacillus* sp. GL1 DNA and the potential cross-reacting microorganisms did not react with the test. Some of the negative control organisms gave a Ct value between 30 and 35, but the Tm was not consistent with the target amplification (80–81 °C). 5 mg/mL mucus and 10 % v/v human saliva did not interfere with the test results at the stated LOD. There were no statistically important Ct differences between the samples with and without interfering substances (t > 2.02, df = 38, p < 0.05).

All of the sites for the reproducibility studies were in 100 % agreement (60/60) for the contrived positive and negative samples. There were no statistically important Ct and Tm differences between the positive tests carried out in the different qPCR instruments (t > 2.00, df = 58, p < 0.05). Biorad CFX Connect, LightCycler 480, StepOne Plus and Xxpress produced Tm values of 80.3 ± 0.2, 80.3 ± 0.1, 80.2 ± 0.2 and 80.2 ± 0.1 °C respectively. Duration of the total assay for 24 samples including pre-analytical processing and analysis was 50 ± 2, 51 ± 3, 55 ± 3 and 42 ± 3 min for Biorad CFX Connect, LightCycler 480, StepOne Plus and Xxpress respectively.

Table 1 compares the results for the developed qPCR method and the RADT, to those of the conventional culture method that is well known as a gold standard for GAS detection [10]. The four different qPCR instruments produced the same test results for the clinical performance evaluation. The qPCR product sequences from the positive 222 throat swabs matched 100 % with *S. pyogenes* strain ATCC 19615 speB gene. PCR inhibition was not detected by means of a lack of internal control (BTL2 gene) amplification for all of the samples.

**Table 1:** Performance characteristics comparison of the developed qPCR method to those of the rapid antigen test (RAT) and the gold standard culture method for detection of GAS from the 687 throat swabs

Results	Culture	RAT	QPCR
Positive	204	114	222
Negative	483	573	465
Prevalence	29.7 %	16.6 %	32.3 %
Sensitivity		69.4 %	100 %
Specificity		100 %	96.4 %
Positive predictive value		100 %	92.9 %
Negative predictive value		84.3 %	100 %

In no case was a throat swab negative by the qPCR method but positive by culture. However, positive results occurred by the qPCR method for some samples that were negative by culture. All of the discordant results by the RADT method versus the results of culture were positive by culture.

## Discussion

Sensitivity and specificity of the DNA amplification based GAS detection tests have already been reported to be 100 and 95 % for PCR [15], 100 and 100 % for qPCR [16] and 100 and 98 % for LAMP [18]. The approximate times required to complete these tests including pre-analytical processing and analysis are as follows: PCR, 3–4 h; qPCR, 1.5–3 h; LAMP, 50–60 min. We showed that the developed qPCR test is rapid (42–55 min), sensitive (100 %) and specific (96.4 %) and therefore can be used to replace both antigen detection and culture for diagnosis of acute GAS pharyngitis. With the speed of the qPCR assay, results can be relayed to health care providers and patients in less than 2 h after the test is ordered.

The high speed of the developed qPCR test was mainly because of the rapid DNA extraction method that can be completed in less than 15 min for 24 samples. The extraction method did not eliminate the possible PCR inhibitors such as collagen [24]. The DNA amplification facilitators, betaine and BSA, were included in the qPCRs to compensate the possible effects of the inhibitors [25]. This application resulted in no PCR inhibition in the clinical performance studies.

In this study, RADT provided the fastest results, but exhibited only 69.4 % sensitivity compared to that of culture. Due to difficulties in the sampling from children, the swab used for RADT was first used to inoculate an agar plate in the clinical performance evaluation studies. This is one of the limitations of the study since some of the bacterial content might have been removed from the swab and affected sensitivity of RADT. The low sensitivity problem of the antigen tests has frequently been reported [13]. If RADTs are performed routinely, culture must be applied to the all RADT-negative specimens. The non-GAS acute pharyngitis accounts for 70–95 % of the cases [1] and only 70 % of the true-positive samples are expected to be detected using RADTs. If culture is performed after RADT, more than 70 % of the patients should have a throat culture.

There are no methods defined in the literature to distinguish GAS carriage from actual streptococcal pharyngitis. All the patients in our study had presented with acute sore throat and up to 12 % of these may actually be carriers [9].

The qPCR product sequences of 18 samples that were culture negative but qPCR positive matched with *S. pyogenes* speB gene. Although throat culture was considered as the gold standard, the detected GAS DNA may be associated with the following: First, some of the patients might have taken antibiotics at the time throat swabs were taken. This would restrict growth on a culture but not the qPCR results. Second, errors of sampling may always be an issue. Third, qPCR may be more sensitive than standard culture. All of the positive patients by qPCR were treated with antibiotics effective against GAS, even though the health care providers responsible for these patients were unaware of the qPCR result. Uhl et al. [17] also reported that all discordant positive results for the qPCR method versus the results of culture were believed to be associated with the disease when they evaluated the GAS test results along with the medical history recorded by the health care provider. On the other hand, the qPCR assay does not distinguish between DNA from viable and non-viable organisms. In order to prevent the false positive results, DNA from the non-viable GAS can be eliminated via pretreatment of the specimens with propidium monoazide [26] or DNase-I [27]. Since the specificity of the developed qPCR test was high enough, we do not recommend application of these pretreatment strategies avoid increase in total analysis time and cost.

The simple DNA extraction using only the Tris–HCl buffer and the low qPCR volume (10 μl) makes the developed assay a very economical alternative for GAS detection. Based on the outcomes of this study, “Republic of Turkey Social Security Institution Health Applications Notification” has recently been updated and “*S. pyogenes* fast PCR test” was added to the reimbursement list [28]. Reimbursement for the *S. pyogenes* fast PCR test is 1.95$ that was calculated based on a testing potential of 2 million tests per year.

## Conclusion

Our rapid, cheap, sensitive and specific qPCR test can replace both antigen detection and culture for diagnosis of acute GAS pharyngitis.
